# KnotProt 2.0: a database of proteins with knots and other entangled structures

**DOI:** 10.1093/nar/gky1140

**Published:** 2018-12-01

**Authors:** Pawel Dabrowski-Tumanski, Pawel Rubach, Dimos Goundaroulis, Julien Dorier, Piotr Sułkowski, Kenneth C Millett, Eric J Rawdon, Andrzej Stasiak, Joanna I Sulkowska

**Affiliations:** 1 Faculty of Chemistry, University of Warsaw, Pasteura 1, Warsaw, Poland; 2 Centre of New Technologies, University of Warsaw, Banacha 2c, Warsaw, Poland; 3 Warsaw School of Economics, Al. Niepodlegosci 162, Warsaw, Poland; 4 Center for Integrative Genomics, University of Lausanne, 1015-Lausanne, Switzerland, SIB Swiss Institute of Bioinformatics, 1015 Lausanne, Switzerland; 5 Vital-IT, SIB Swiss Institute of Bioinformatics, 1015 Lausanne, Switzerland; 6 Faculty of Physics, University of Warsaw, Pasteura 5, Warsaw, Poland; 7 Walter Burke Institute for Theoretical Physics, California Institute of Technology, Pasadena, CA 91125, USA; 8 Department of Mathematics, University of California, Santa Barbara, CA 93106, USA; 9 Department of Mathematics, University of St. Thomas, Saint Paul, MN 55105, USA

## Abstract

The KnotProt 2.0 database (the updated version of the KnotProt database) collects information about proteins which form knots and other entangled structures. New features in KnotProt 2.0 include the characterization of both probabilistic and deterministic entanglements which can be formed by disulfide bonds and interactions via ions, a refined characterization of entanglement in terms of knotoids, the identification of the so-called cysteine knots, the possibility to analyze all or a non-redundant set of proteins, and various technical updates. The KnotProt 2.0 database classifies all entangled proteins, represents their complexity in the form of a knotting fingerprint, and presents many biological and geometrical statistics based on these results. Currently the database contains >2000 entangled structures, and it regularly self-updates based on proteins deposited in the Protein Data Bank (PDB).

## INTRODUCTION

In recent years it has been found that protein chains can be entangled and form knots, slipknots, and other topological structures, and much effort has been invested into identifying such structures in the PDB. The first examples of knots in proteins were found in 1994 (1), and many more have been identified in recent years (2–5). Slipknots, defined as chains which contain a knotted subchain, however are unknotted as a whole, were found in proteins in 2007 (6), and their systematic analysis was initiated in (7,8). Knots in closed subchains of proteins can also be defined by taking into account disulfide bonds or ions. In addition, the concept of knotoids—which generalize knots to the case of chains with open ends—has been recently shown to be particularly well suited to characterize entangled proteins (9,10). Yet another interesting class of entangled proteins are those with the so-called cysteine knots (11,12); note that cysteine knots are not ‘knots’ in the topological sense, although their entangled pattern is also of interest to researchers. KnotProt 2.0 collects all proteins with these structures, characterizes their entanglement using appropriate mathematical tools (10,13,14), and assembles relevant biological information and various statistics.

Considerable interest has arisen recently around entangled proteins for a variety of reasons. First, it is believed that the presence of entangled structures in proteins is not accidental, and therefore understanding their function is an important challenge. Second, recent work shows nearly perfect conservation of knotting fingerprints in some families whose members differ by hundreds of millions years of evolution (arising from distant organisms) and which possess a low sequence identity (8). Moreover, based on knotting fingerprints, it was shown that the locations of active sites in proteins are correlated with the positions of the knot cores (15,16). These findings imply that a detailed representation of protein topology can be crucial for understanding their biological role (17). The KnotProt 2.0 database makes entanglement data easily available and will help researchers to understand the biological role of entanglement in proteins.

Usually it is impossible to determine by eye whether a given protein chain forms a knot, a slipknot, or a knotoid. Therefore, more involved mathematical tools, such as polynomial knot invariants, are used to detect such structures. The KnotProt 2.0 database takes advantage of such tools, and, among many features, characterizes knots by means of matrix diagrams called ‘knotting fingerprints’ which encode information about the knot type of each subchain of a protein backbone. The structure of the knotoids is also represented by projection globes/maps.

**Figure 1.**
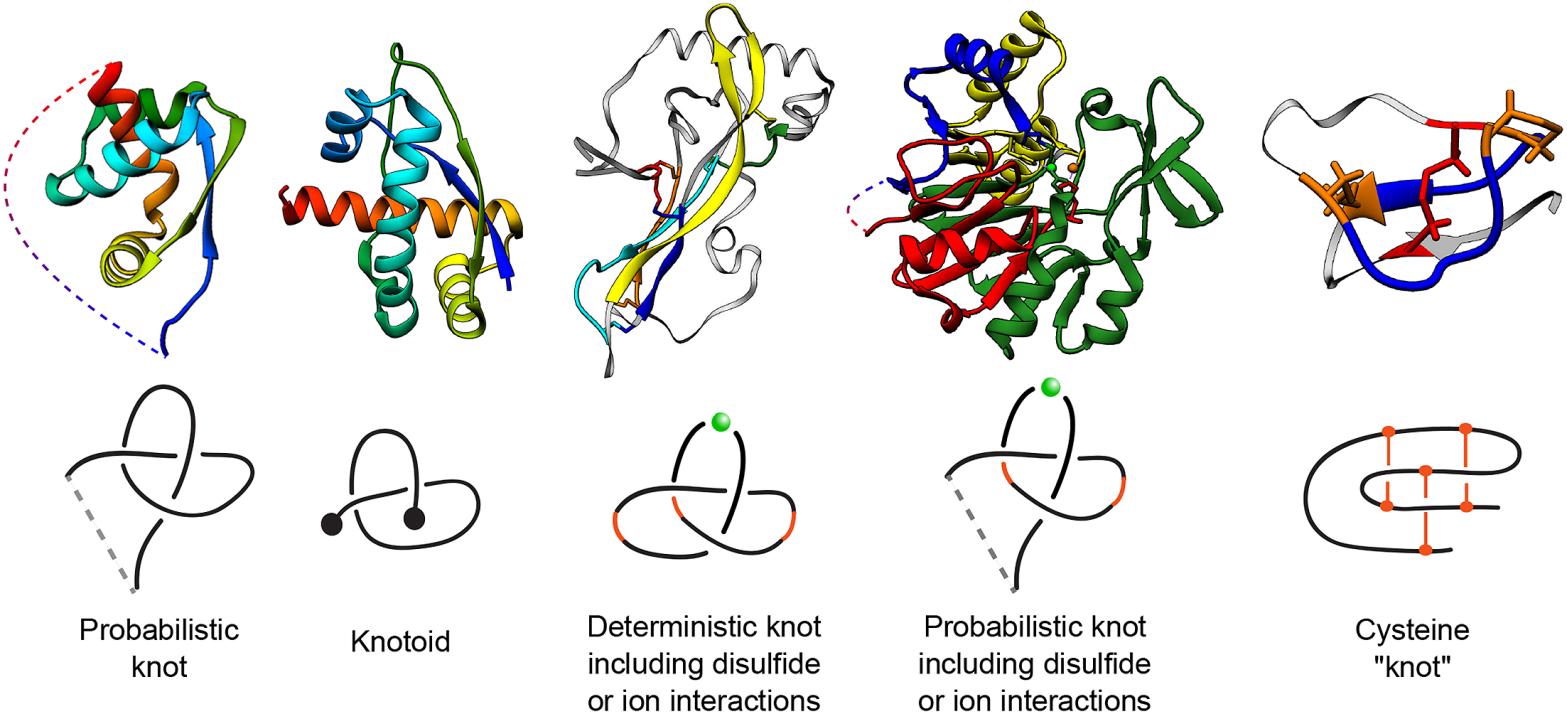
Proteins that represent various classes of entangled structures identified by KnotProt 2.0 (top row, structures with PDB codes, from left to right: 2EFV, 2RH3, 1AOC, 2P4Z, 2ML7) and their schematic representation (bottom row). Probabilistic knots are identified based on the protein backbone chain; the dashed line denotes a possible chain closure. All the structures with probabilistic knots are subjected to the knotoid analysis. Knotoids provide a refined characterization and classification of open chains, which depends, however, on the choice of the projection plane. Deterministic knots are detected based on all possible combinations of covalent bonds or interactions via ions with the protein’s backbone. In the case of probabilistic covalent and ion-based knots, the chain is closed based on the methods established previously for knotted proteins. The green bead in the schematic depiction denotes the ion, while the orange stripes denote the covalent connections (e.g. disulfide bonds). For structures with covalent bridges, consecutive parts of the chain constituting the knot are colored red to blue. The parts of the chain not building the knot are marked with light grey. The rightmost structure is a cysteine knot, which in fact is not a mathematical knot (for more details see Figure 11); in this example, the loop (blue) is closed by two disulfide bridges (orange) and pierced by the third disulfide bond (red).

Moreover, note that the knots in proteins analyzed to date were defined by taking into account only the protein backbone (or its subchains), whose open ends needed to be connected to identify a knot type. Since there is no unique way of connecting the open ends, probabilistic methods are used to characterize such connections and so we call the resulting knots ‘probabilistic’. However, one can also define knots formed by closed subchains of a protein chain, once disulfide bonds or interactions via ions are taken into account. Such closed subchains do not have open ends, so the knots that they form are unique, and we call them ‘deterministic’. KnotProt 2.0 also analyzes and classifies these deterministic knots. A schematic representation of probabilistic and deterministic knots, knotoids, and the so-called cysteine knots, is shown in Figure 1. More details on how these structures are defined and detected are discussed in what follows.

The KnotProt 2.0 database also presents extensive statistics about knots (including those formed by disulfide bonds or interactions via ions), slipknots, and knotoids identified in proteins. Proteins with such structures can be sorted based on their biological function, molecular tags, family association, or type of fold, as well as based on geometric data such as knotting patterns, knot and slipknot lengths, depths, etc. Interestingly, the KnotProt 2.0 analysis reveals that proteins with knots, slipknots, and knotoids can be classified into a few distinct topological motifs, represented by particular patterns within the knotting fingerprints. This data can be used, for example, to find entangled proteins with a given homological sequence or with a similar structure, or to find entangled proteins which perform a particular biological function. The topological characterization of proteins rich in disulfide bond or interactions via ions should help to explain their thermodynamical or mechanical stability, as well as their reasons for misfolding. Moreover, proteins with cysteine ‘knots’ are targets for commercial and medical use (including anti-HIV, anti-bacterial and insecticidal activity) (18–20). Representing the entanglement in proteins via knotoids reveals more details about their complexity, and should be interesting objects of study for mathematicians. The KnotProt 2.0 database and all its statistics are automatically updated every Wednesday, immediately after new structures are deposited in the PDB.

As an additional feature, KnotProt 2.0 has a submission server mode that enables users to analyze structures and generate knotting and knotoid fingerprints of uploaded proteins. It is also possible to upload and analyze a whole set of structures, e.g. to analyze the evolution of a knot along a folding or unfolding trajectory.

In more detail, new features of KnotProt 2.0 (as compared to the first version of the KnotProt (21)) include:

- the possibility to analyze chains which include disulfide bonds and interactions via ions for both probabilistic and deterministic knots, in addition to those formed purely by the whole protein backbone,
- the characterization of entangled structures in terms of knotoids,
- the analysis of cysteine knots,
- the choice of analyzing all protein structures in the database or just a non-redundant set,
- the implementation of faster and more efficient algorithms to detect non-trivial topologies and to compute the topological fingerprints; these improvements are also implemented in the submission server.

## MATERIALS AND METHODS

### Knot detection and the knotting fingerprint

To detect a knot formed by a protein chain—or one of its subchains, possibly defined by including disulfide bonds or interactions via ions—we project it on a plane and compute polynomial knot invariants, such as the Alexander and HOMFLY-PT polynomials. In the case of probabilistic knots, to define a closed chain the endpoints are connected via points on a large sphere surrounding the structure. Since the knot type depends on the choice of these connecting points, we determine the resulting knots for a large set of equally distributed points on the sphere, and to each resulting knot type we assign its probability (i.e. the fraction of points on the sphere that give rise to the same knot type). Furthermore, we characterize the knotting within the protein in more detail using the knotting fingerprint, which is a matrix diagram that encodes information about the knot type of each subchain of a protein backbone. The above methods are essentially the same as the previous version of the KnotProt database, and are presented in detail in (21).

### Knotoids, their detection and visualization

**Figure 2.**
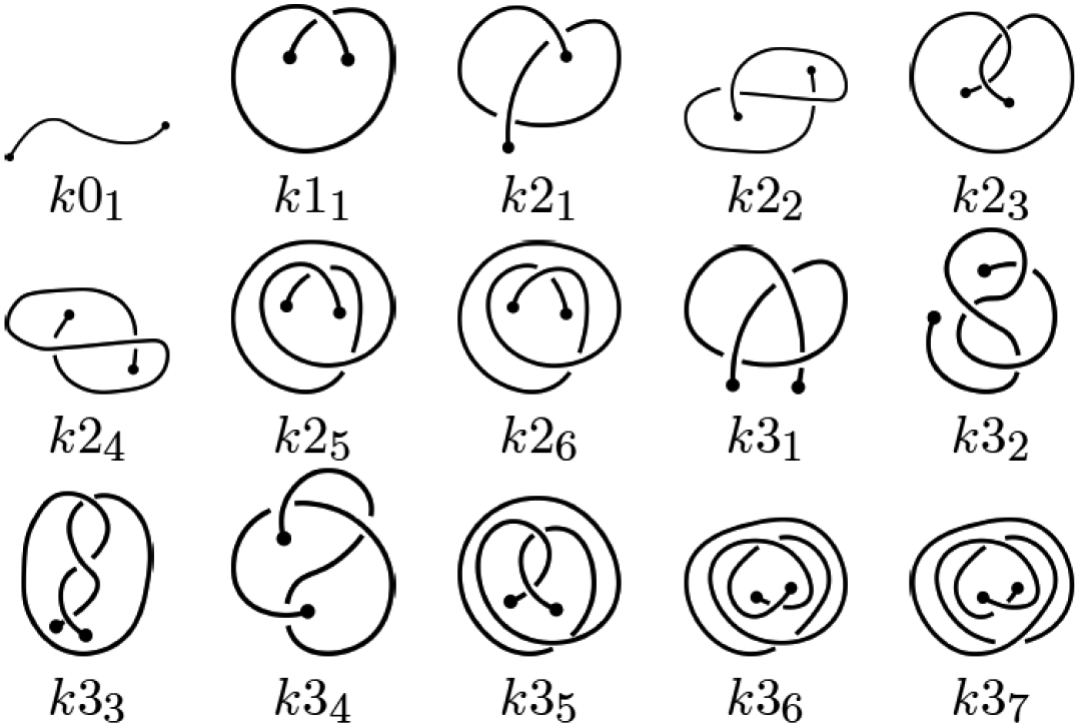
A table of knotoids through three crossings. The letter *k* in the notation refers to a ‘knotoid’, and the first number indicates the minimal number of crossings of the given knotoid. The second number (written in subscript) is needed to distinguish different knotoids with the same minimal number of crossings. Here this second number corresponds to the notation of corresponding knotoids applied in Knoto-ID (22).

**Figure 3.**
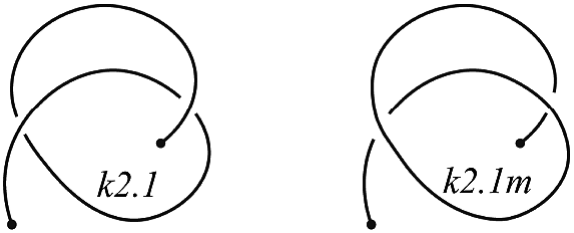
An example of the knotoid *k*2.1 and its mirror reflection, denoted *k*2.1*m*.

Knotoids are a novel mathematical concept which turns out to be well-suited to characterize entanglement in proteins (9,10). A knotoid is a projection of an open-ended 3D curve onto a plane or a sphere, with additional information about which strands of the curve pass over and under at cross-over points. In what follows we consider knotoids defined via a projection on a plane (called planar knotoids), which encode more detailed information about a given structure than projections on a sphere (called spherical knotoids) (10). The topology of the knotoid diagram is preserved under Reidemeister moves that do not involve endpoints of a diagram (see e.g. (9)); in particular, the endpoints are not allowed to cross any of the arcs of the diagram during the Reidemeister moves. Knotoids that involve up to three crossings, together with their notation, are shown in Figure 2. One can also consider a mirror reflection of a knotoid, which is defined by inverting all crossings of the original knotoid diagram, as shown in Figure 3. In the notation, mirror reflections are denoted by an additional symbol ‘m’.

Similar to the case of knots, knotoid types can be determined by knotoid invariants. To determine planar knotoid types we use the Turaev loop bracket polynomial (9,23). To shorten computational time, a variation of the KMT algorithm is applied (once the projection plane has been chosen and the positions of the two endpoints on this plane are fixed).

**Figure 4.**
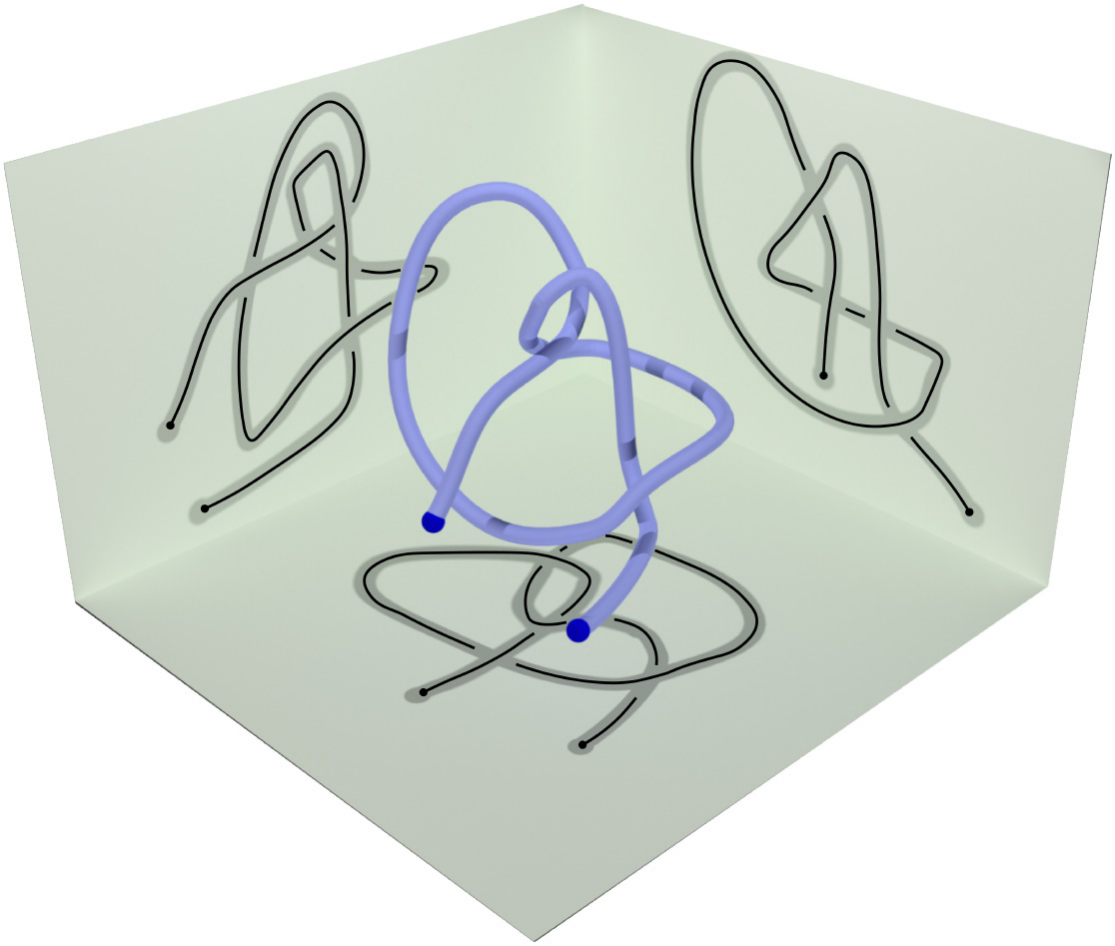
A knotoid is defined via a projection of an open 3D curve onto a plane. Its knotoid type depends on the choice of the plane.

An obvious advantage of knotoids is that they classify and encode information about open chains, without the need to connect their endpoints. However, since a knotoid is defined via a projection on a plane, its knotoid type depends on the choice of such a plane (as shown in Figure 4) and we rely on probabilistic technqiues analogous to our work with knots. The classification via knotoids is more refined than that of knots, and thus they provide additional details in characterizing entangled proteins.

KnotProt 2.0 detects and analyzes knotoid types within the backbone chain formed by the C_α_ atoms of the protein. Since the knotoid type of a protein chain (or subchain) depends on the projection direction, we consider the probability distribution of knotoid types over 100 uniformly distributed projection directions (a larger number would improve precision, but makes computations slower). The knotoid type with the highest probability in the distribution is then called dominant and is used as the representative of the distribution.

To present the knotoid structure of proteins we use two visualizations, projection globes/maps and fingerprint matrices; both are computed using Knoto-ID (22).

**Figure 5.**
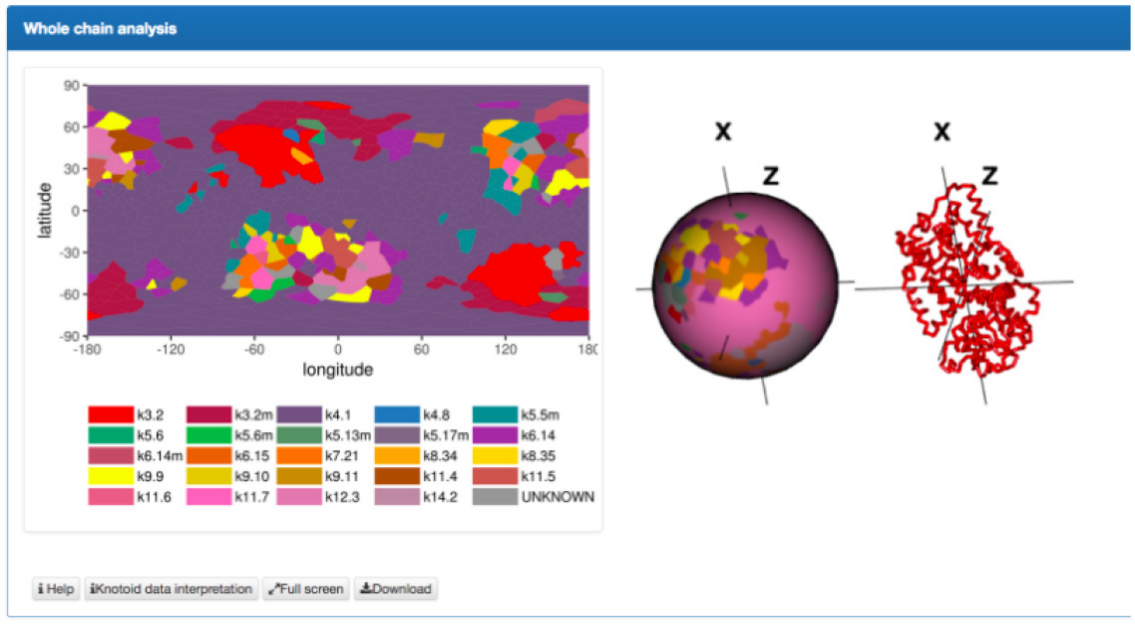
Projection globe/map. Each point in this map represents a projection direction that defines a knotoid, and its color denotes the resulting knotoid type.

The projection globe/map is a map that shows the knotoid types obtained when the full protein chain (these maps are not included for subchains) is projected in all directions on a sphere. The color of each point represents the knotoid type resulting from projecting the full protein chain in the given direction. The resulting map typically contains several colored regions. The dominant knotoid type corresponds to the region that occupies the largest total area in the map, which is usually easily identified. An example of the projection map is shown in Figure 5.

**Figure 6.**
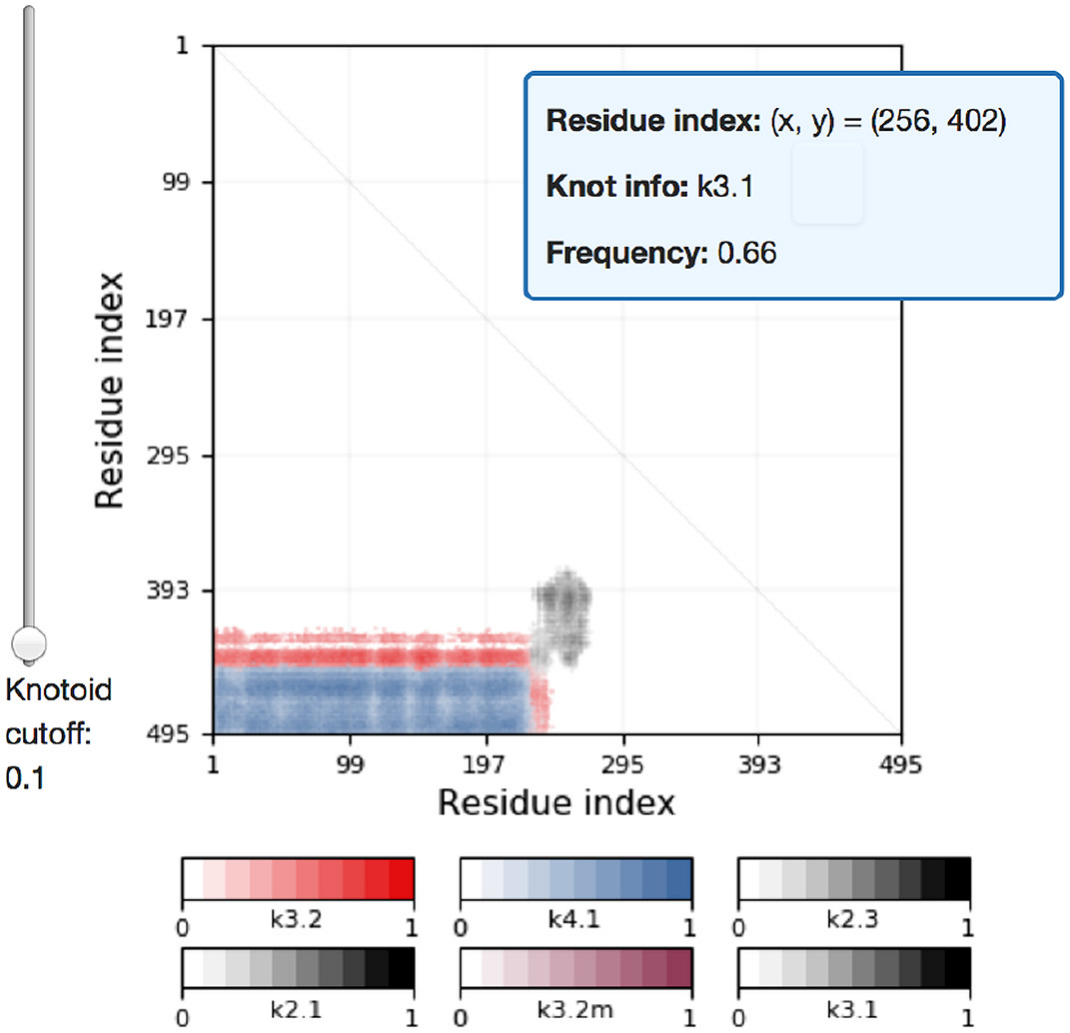
An example of a fingerprint matrix. Each point in the matrix represents a subchain of the protein chain (whose beginning and end correspond to *x*- and *y*-coordinates of this point), and its color denotes the dominant knotoid type of the corresponding subchain. The assignment of colors to the knotoid types is provided in the key. After pointing the cursor at a point corresponding to some knotoid, its *x*- and *y*-coordinates, knotoid type, and frequency are shown in a blue box.

Additionally (knotoid) fingerprint matrices are presented. These matrices are analogous to knotting fingerprints for knotted proteins (21). In order to construct a fingerprint matrix we consider all subchains of a given protein chain. A choice of a subchain is represented by a point in a (lower-triangular) matrix diagram, whose *x*- and *y*-coordinates correspond to sequential numbers of amino acids that define the beginning and the end of the subchain. In particular, the whole chain is represented by the bottom left corner of the matrix. Each point in the matrix is assigned a color that denotes the dominant knotoid type of the corresponding subchain. The correspondence between colors and knotoid types is indicated in a provided color key. In the fingerprint matrix we also identify the ‘knotoid’s core’, i.e. the shortest subchain whose knotoid type is the same as the knotoid type of the whole chain. An example of the fingerprint matrix is shown in Figure 6.

### Knots formed via disulfide bonds and ion interactions

**Figure 7.**
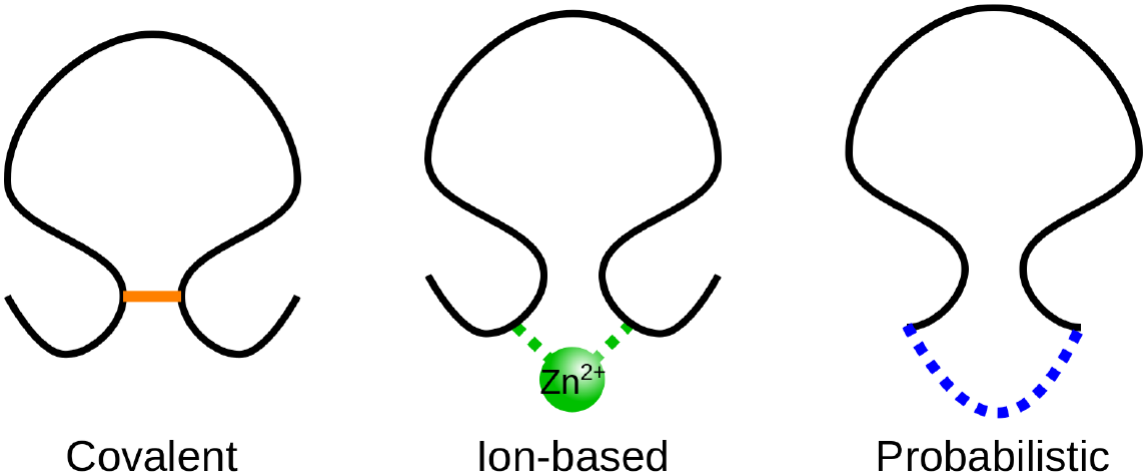
Schematic presentation of the three types of interactions included in the KnotProt 2.0 database.

**Figure 8.**
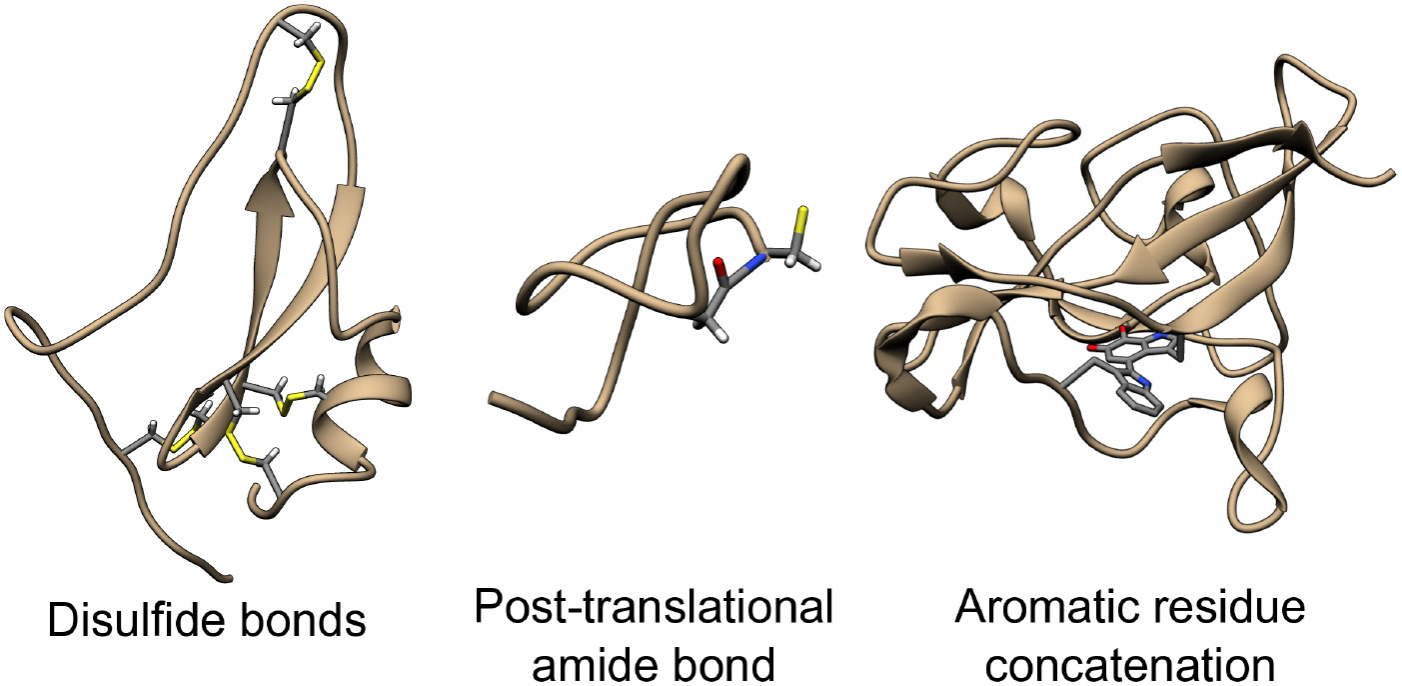
Examples of protein structures with various covalent bonds between non-sequential residues. A disulfide bond (within hydrolase inhibitor, PDB code 2LFK), a post-translational amide bond closing the loop (within replication inhibitor, PDB code 1RPB), and a concatenation of aromatic side chains (within oxidoreductase, PDB code 3C75). The atoms of joined residues are shown and colored.

**Figure 9.**
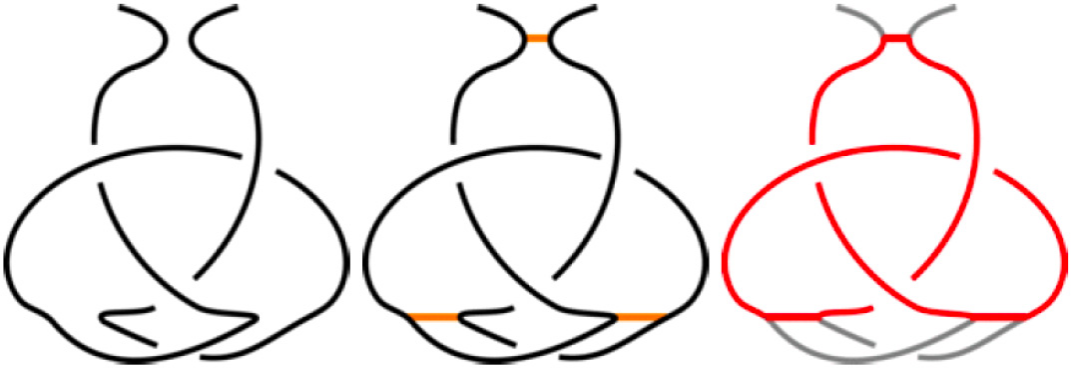
A schematic presentation of an unknotted protein with a knotted, covalent loop. The protein backbone is presented in black, and the covalent bridges in orange. The knotted loop is highlighted in red.

Usually the term ‘knotted protein’ refers to the situation where the backbone of the protein forms a knot after some sort of chain closure; in our work, the closure is not uniquely specified, so we refer to such knots as ‘probabilistic’, see Figure 7 (right). In defining such knots, the choice of the backbone is well-justified since the backbone is composed of strong covalent bonds joining carbon and nitrogen atoms. However, some residues may be also joined non-sequentially either by ions, by disulfide bonds, by post-translational amide bonds, or as a concatenation of the side groups. Such connections are shown schematically in Figure 7, and in more detail in Figure 8. The existence of such bridges and bonds allows one to define inter-protein loops, which may, in principle, be knotted as well. The identification of knots formed by such loops provides a more detailed description of the protein’s topology. If such loops are closed (and do not involve the protein endpoints), the knots they form are mathematical knots and we call them deterministic. Note, in particular, that a protein may have an unknotted backbone, while some of its other loops may be knotted. Such a situation is shown schematically in Figure 9. KnotProt 2.0 detects both probabilistic knots formed by the whole chain, as well as deterministic knots, in this case analyzing all possible loops in a protein formed by disulfide bond or ion interactions. Moreover, KnotProt 2.0 detects probabilistic knots (as explained earlier) in open protein chains which include disulfide or ion bonds.

**Figure 10.**
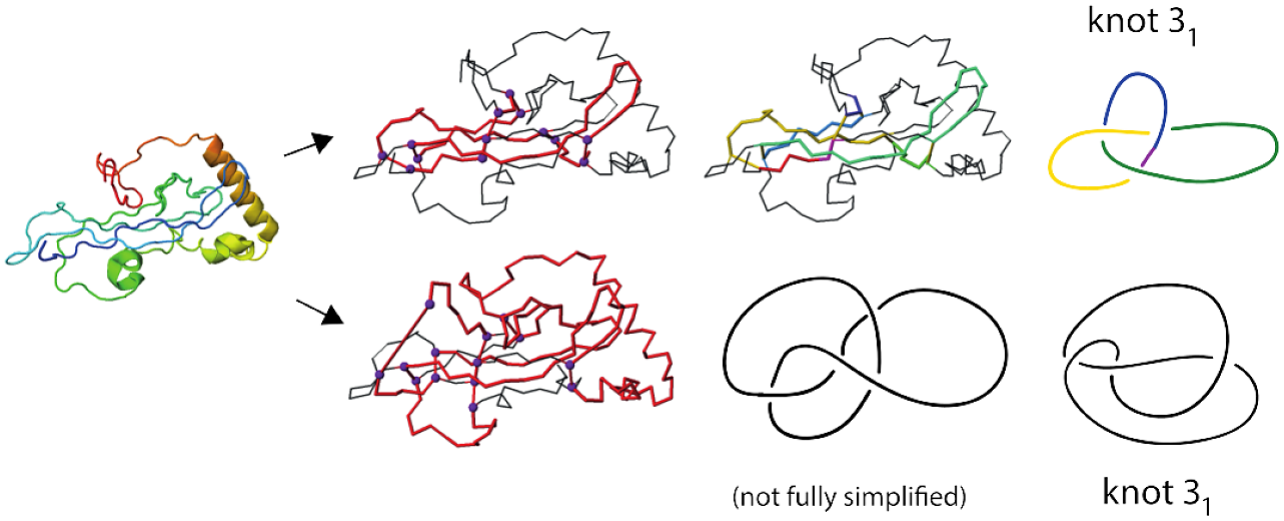
Cartoon representation of the clotting protein from the horseshoe crab, left. The structures on the right show two different trefoil knots identified based on disulfide bonds and the protein backbone. The magenta spheres indicate the positions of the cysteines which form the disulfide bonds. The red color indicates the location of the cysteine knot.

As an interesting example of a deterministic knot, in Figure 10 we show two of the three identified trefoil knots in a coagulogen, the clotting protein from the horseshoe crab: a structural homologue of nerve growth factor (PDB code 1aoc, chain A). The topological characterization of this protein computed by KnotProt 2.0 uncovers its hidden complexity.

### Cysteine knots

**Figure 11.**
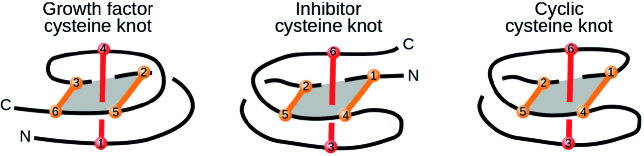
Three types of cysteine knots identified in proteins. The loop-forming bridges are shown in orange, and the piercing bridge in red. The ‘N’ and ‘C’ letters denote the chain termini. The numbers in the beads representing the cysteines illustrate the sequential order of the cysteine residues.

The so-called cysteine knots form yet another interesting class of topological structures in KnotProt 2.0. Cysteine knots are formed by three disulfide bridges, such that two of them form a covalent loop, which is pierced by the third one. There are at least three classes of cysteine knots, differing in the bridge arrangement and the connection of the termini, see Figure 11.

**Figure 12.**
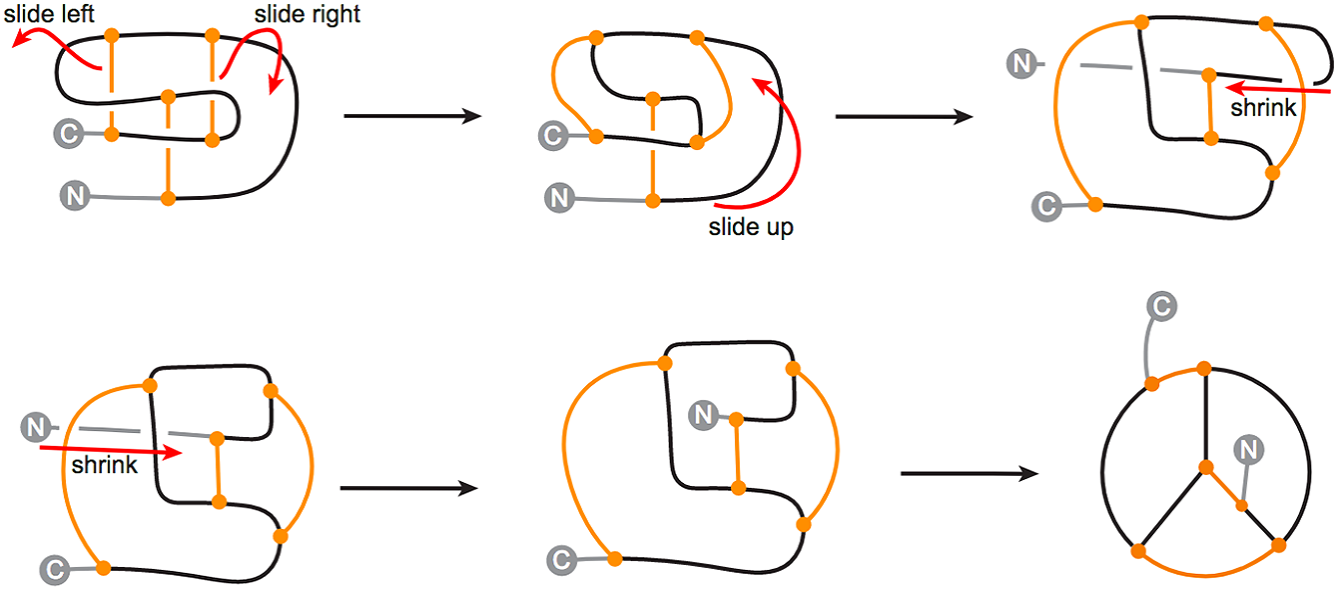
The continuous transformation of growth factor cysteine into a simpler form in which all the closed, covalent loops (circular paths) are clearly unknotted. The orange stripes denote the bridges and the blue dashed line denotes the chain closure. The gray arrows indicate the parts of the chain being pushed in subsequent steps. The ‘N’ and ‘C’ letters denote the chain termini. The protein with the termini connected is equivalent to the bottom-middle scheme. A similar operation may also be performed in the case of inhibitor and cyclic cysteine knots (see the ‘Cysteine knots’ description on the KnotProt 2.0 website).

It should be stressed that cysteine knots do not form knots in the topological sense, and so their name may be misleading. In particular, each covalent loop (circular path following covalent bonds) is unknotted. This can be seen directly in the continuous deformation of the motif of Figure 12. Moreover, it is not hard to see that even the connection of the termini does not introduce any knotted, closed loop. This idea can be made mathematically precise by treating the motif as a spatial graph, which in such case does not feature any knotted cycle (i.e. is not intrinsically knotted/linked). Nevertheless, as the ‘cysteine knots’ are topologically complex motifs, which have become known world-wide, the KnotProt 2.0 database includes a self-updating list of cysteine knots. Two lists are available: a list of all structures classified as cysteine knots and a list of non-redundant representants. The lists may be found in the top of the ‘Search’ tab, as well as in the ‘Read more’ section.

The list of cysteine knots identified in KnotProt 2.0 includes their PDB code with the chain name, the loop, and the piercing bridge. The covalent, pierced loop is determined by the indices of the two cysteine bridges. However, it is also convenient to list explicitly the subchains connected by the bridges. Such subchains are encoded by specifying the indices, connected by the ‘-c-’ (chain) separator. Similarly, the ‘-b-’ denotes a connection by a bridge. Therefore, a covalent loop may be represented, for example, as ‘4-c-11-b-23-c-21’. Such a string denotes that the covalent loop is formed by the subchains Cys4→Cys11 and Cys21→Cys23, and is connected by the bridges Cys11–Cys23 and Cys4–Cys21 (with the second bridge being implicit). Similarly, the piercing bridge is described, for example, as ‘17-b-29’, meaning the disulfide bridge is between Cys17 and Cys29. Clicking an entry displays the page which shows all data on the non-trivial knot-like motif in proteins.

**Figure 13.**
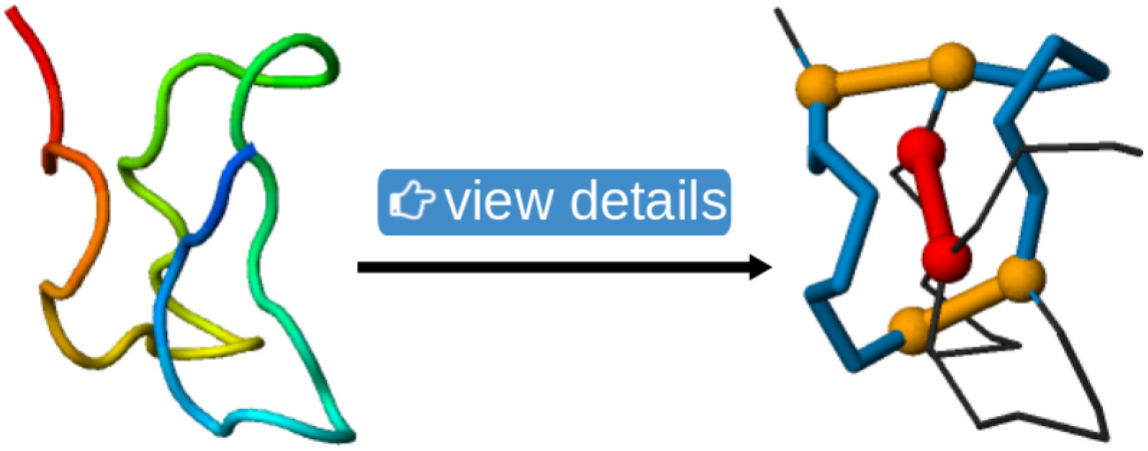
The result of the ‘View details’ button on the structure visualization. The loop-forming subchains are in blue, the loop-forming bridges are in orange, and the loop piercing bridge is in red.

Each protein with a cysteine knot listed in the database has a separate tab with the structural description of its cysteine knot motif. In particular, the tab contains a table row with the loop and the piercing bridge. Clicking the ‘View details’ button shows the loop in the visualization of the structure, and on the sequential representation of the protein. The loop-forming arcs are in blue, the loop-forming bridges are in orange, and the loop-piercing bridge is in red, see Figure 13.

Finally note that the definition of the cysteine knot invokes only the mutual sequential and spatial arrangement of the bridges, but does not impose any condition on the sequential distance between the bridges. Therefore, KnotProt 2.0 classifies as a cysteine knot any kind of triple of disulfide bonds, independent of the size of the motif.

### Proteins in the KnotProt 2.0 database

The database contains the topologically non-trivial structures found in the entire RCSB PDB database, including crystal, NMR, and EM structures, and also includes structures with missing atoms. For proteins missing atoms, the gap in the main chain is filled with a straight line segment. The database self-updates every Wednesday. All of the analyzed (both entangled and not entangled) structures are available as a list of entries. More details about structures identified in KnotProt 2.0 are provided in the ‘Results’ section below.

### Database technicalities

The database is written in Python with the Flask framework dynamically generating HTML pages using Apache2 with WSGI. The data is stored using a SQL database. Information about the proteins is downloaded from the PDB using RESTful services, and the PFAM and EC data using the SIFTS service. The whole service is installed on multicore Linux nodes and uses an asynchronous message queue for a reliable and efficient distribution of computing tasks to all GPU and CPU compute nodes.

### SORCER Modeling Framework (SMF)

The SORCER Modeling Framework (SMF) developed in (24,25) is applied to accelerate various computations. In particular, the calculation of knotoids using the Knoto-ID package (22) is a multistage process that, in particular for larger proteins, takes a significant amount of time to compute. The computation of knotoids can take anywhere from minutes to several weeks for a single protein chain. The KnotProt 2.0 database at the time of article submission included over 1500 protein chains, whose analysis required an efficient computational environment. Although SORCER and the SORCER Modeling Framework were initially designed for use in engineering, in particular in Trans- and Multidisciplinary Design Optimization (MDO), this platform can be easily adapted to serve in any distributed computing application. For our computations, SMF was used to integrate the Knoto-ID solvers and post-processing scripts, and to distribute the computations across a network of desktops and clusters.

## DATABASE INTERFACE AND DATA PRESENTATION

The database interface is intuitive and—despite many new features and entanglement types—analogous to the previous version of KnotProt (21). There is a ‘Read more’ online section which explains how to conduct various actions (e.g. to search or to browse the database, analyze various structures, interpret knotting data, submit files, etc.), and also ‘Help’ buttons are available in various places in the webpage for the user’s convenience. Nonetheless, let us briefly summarize the main features of the database interface.

From the menu on the homepage a user can choose to ‘Browse database’, and then choose a tab to view the list of proteins with knots or knotoids. The list of knotoids is a new feature of KnotProt 2.0. Each list can be shown in several views: either the basic one (which includes the entanglement type, PDB code, and the name/origin of the protein), or raw data, or a view including the fingerprint matrix.

The second main window is ‘Search database’. Here one can choose to list various classifications (based on entanglement type, or schematic fingerprint, or core and tail lengths) of knots or knotoids (which is a new option). One can also view classifications based on molecular keywords, PFAM family identifiers, EC nomenclature, or CATH classification. Furthermore, new important options enable users to choose browsing either all entries or a non-redundant set of proteins, as well as probabilistic or deterministic knots. Finally, choosing ‘Cysteine knots’ (also a new option) button shows a list of such structures.

Once a particular structure is chosen (either from the ‘Browse’ list or the ‘Search’ menu), the structure is shown in a separate window, where several views are possible: the basic knotting data, knotted loops (new option), or knotoid analysis (new option). Each of these options includes the fingerprint (knot or knotoid) matrix, JSmol visualization, the table with knot or knotoid details (the core range, length, tails range, etc.) and the sequential structure. Furthermore, one can choose to see the detailed ‘Chain information’, as well as ‘Similar chains (by sequence)’. Analogous details are shown for structures with cysteine knots.

Other sections of the database include the server option (‘Process my structure’, described in the next section) that enables users to analyze uploaded structures, and a detailed help section (‘Read more’), which, in particular, includes database statistics (briefly summarized in the ‘Results’ section below).

## SERVER—ANALYZE UPLOADED (BIO)POLYMERS

Apart from the database, KnotProt 2.0 gives users the unique opportunity to upload and then analyze various structures. The user can upload the file in two formats: PDB or an ASCII file consisting of atom indices and their *X, Y*, and *Z* coordinates. The output is presented in the same manner as in the database, including the fingerprints and downloadable files. The results are stored for two weeks.

## RESULTS

The KnotProt 2.0 database collects information about entangled protein structures that form probabilistic and deterministic knots, slipknots, knotoids, and cysteine knots. The database is updated weekly, so the number of such structures is growing, and many new interesting proteins are regularly identified. For example, some novel structures that have been found include knotted mitochondrial proteins, the most deeply embedded protein knot discovered so far, and knotted membrane proteins. As another example, very recently a new knotted protein—flagellin derivative in complex with the NLR protein NAIP5 (PDB code 5yud)—has been found.

General information about the identified structures is available in the ‘Database statistics’ section of the database. As of September 2018, out of the total of above 225.000 chains from the PDB, KnotProt 2.0 has identified 1549 knotted entries, 529 knotted chains, and 1020 chains that form slipknots. There are 1506 entries that form non-trivial knotoids: 1023 separate protein chains which form non-trivial knotoids and 483 chains which form trivial knotoids but which have subchains that form non-trivial knotoids (i.e. form ‘slipknotoids’). Furthermore, 608 chains with knots based on disulfide bonds or ion interactions have been found, and 968 cysteine knots. All this data is regularly updated once new entangled structures are identified.

The statistics section also reports the oldest and the newest proteins with knots, slipknots, knotoids, and knotoids with slipknots, identified by KnotProt 2.0.

Various entangled structures identified by KnotProt (and other databases) are also described in more detail in recent review articles (5,26).

## APPLICATIONS

We now discuss some potential applications of the KnotProt 2.0 database. Note that several such applications were discussed in detail already in the context of the previous version of the KnotProt, and they include: trajectory analysis, analysis of knots and slipknots from the viewpoint of thermodynamics processes, analysis of proteins stretched by the AFM or optical tweezers, and trajectory analysis to find the optimal direction of pulling the protein to untie it (21). All of these applications are still relevant for KnotProt 2.0. In what follows we discuss in more detail some new applications of the updated version of the database.

One new application of KnotProt 2.0 is related to protein stability. It is well known that cysteine bonds introduce additional stability to protein structures (19,27,28); note that in these works various applications of proteins with such bonds are mentioned, which are also relevant for the structures in KnotProt 2.0. Moreover the analysis of proteins with links (which can be found in the LinkProt database (29)) also revealed that non-trivial topologies introduce additional stability (30) (e.g. Cerato-platanins has been shown to be stable up to 76°C (31); Fungal endoglucanase (1hd5) is stable after heating in 60°C and in pH between 3.0 and 11.0 (32), and retains 45% of its activity after incubating for 5 min at 95° C (32); the animal endoglucanase (1wc2) withstands heating for 10 min at 100° C without irreversible loss of activity (33)). Yet another example are the so-called cyclotides (34). It has been shown that the circular protein backbone and knotted arrangement of disulfide bonds make the cyclotides also exceptionally stable (19). Following the example given on the cyclotides webpage, ‘In their native medicine uses, for example, the peptides are boiled when the plant Oldenlandia affinis is used to make a tea that women ingest to accelerate childbirth’ (35). These peptides are exceptionally stable to enzymatic degradation (19,20). Such peptides, due to their stability, are commonly used as templates in pharmaceutical applications (including anti-HIV, anti-bacterial and insecticidal activity (19,20)).

In view of the examples mentioned above, it follows that the topological characterization of proteins rich in disulfide bond or interactions via ion in KnotProt 2.0 should help in explaining their thermodynamical or mechanical stability, as well as their reasons for misfolding.

Furthermore, proteins with probabilistic or deterministic knots formed via cysteine bonds or interactions via ions identified in KnotProt 2.0 could provide new templates for commercial and medical applications.

## ON-LINE DOCUMENTATION

The database is supported by extensive on-line documentation which includes: detailed descriptions of the detection methods for probabilistic and deterministic knots, knotoids, and cysteine knots; instructions for how to search, browse, analyze, and interpret the results from the database; and a description of the server options. Database statistics, as well as lists of all and non-redundant cysteine knots, are also provided.

## COMPARISON WITH OTHER DATABASES

To our knowledge KnotProt 2.0 is the first database which contains comprehensive information about cysteine knots, ion based knots, and knotoids formed by protein chains. Our database collects more information than e.g. the KNOTTIN database (http://www.dsimb.inserm.fr/KNOTTIN/), or the cyclotides webpage (http://www.cyclotide.com/cybase.html), which provided some information about cysteine knots, but now is inactive. In comparison to other databases such as Protein Knots (http://knots.mit.edu/) or pKNOT (http://pknot.life.nctu.edu.tw), KnotProt 2.0 is the only regularly self-updating and manually-corrected database, and it organizes and analyzes a much larger set of motifs. Furthermore, KnotProt 2.0 collects and presents a great deal of biological and other data (which is not available as a unified package in other databases).

## SUMMARY

KnotProt 2.0 is an extensively updated version of the database. It collects information about proteins with knots and slipknots (as in the original version), as well as with knotoids, cysteine knots, and novel structures with knotted loops formed by disulfide or ion bonds. In this version, a user can browse either all structures or a non-redundant set, and analyze both probabilistic and deterministic knots. Information about the entangled structure of a given protein is presented in the form of a fingerprint matrix, together with other geometric data. The database also provides information about related biological features and various classifications of proteins, and is automatically updated weekly. Furthermore, the database includes the server option that enables users to analyze uploaded structures. This is the only database which includes such extensive information and it should find applications in biophysics, bioinformatics, biochemistry, and other fields.

## References

[1] Mansfield M.L. Are there knots in proteins. Nat. Struct. Biol. 1994; 1:213–214. 7656045 10.1038/nsb0494-213

[2] Taylor W.R. A deeply knotted protein and how it might fold. Nature. 2000; 406:916–919. 10972297 10.1038/35022623

[3] Virnau P., Mirny L.A., Kardar M. Intricate knot in proteins: Function and evolution. PLoS Comput. Biol. 2006; 2:1074–1079. 10.1371/journal.pcbi.0020122 PMC1570178 16978047

[4] Bölinger D., Sulkowska J.I., Hsu H.-P., Mirny L.A., Kardar M., Onuchic J.N., Virnau P. A Stevedore’s protein knot. PLoS Comput. Biol. 2010; 6:e1000731. 20369018 10.1371/journal.pcbi.1000731 PMC2848546

[5] Sulkowska J.I., Sulkowski P. Entangled proteins: knots, slipknots, links, and lassos. The Role of Topology in Materials. 2018; Springer 201–226.

[6] King N.P., Yeates E.O., Yeates T.O. Identification of rare slipknots in proteins and their implications for stability and folding. J. Mol. Biol. 2007; 373:153–166. 17764691 10.1016/j.jmb.2007.07.042

[7] Sulkowska J.I., Sulkowski P., Onuchic J.N. Jamming proteins with slipknots and their free energy landscape. Phys. Rev. Lett. 2009; 103:268103. 20366349 10.1103/PhysRevLett.103.268103

[8] Sulkowska J.I., Rawdon E.J., Millett K.C., Onuchic J.N., Stasiak A. Conservation of complex knotting and slipknotting patterns in proteins. Proc. Natl. Acad. Sci. U.S.A. 2012; 109:E1715–E1723. 22685208 10.1073/pnas.1205918109 PMC3387036

[9] Goundaroulis D., Dorier J., Benedetti F., Stasiak A. Studies of global and local entanglements of individual protein chains using the concept of knotoids. Sci. Rep. 2017; 7:6309. 28740166 10.1038/s41598-017-06649-3 PMC5524787

[10] Goundaroulis D., Gügümcü N., Lambropoulou S., Dorier J., Stasiak A., Kauffman L. Topological models for Open-Knotted protein chains using the concepts of knotoids and bonded knotoids. Polymers. 2017; 9:444. 10.3390/polym9090444 PMC6418563 30965745

[11] Craik D.J., Daly N.L., Bond T., Waine C. A unique family of cyclic and knotted proteins that defines the cyclic cystine knot structural motif. J. Mol. Biol. 1999; 294:1327–1336. 10600388 10.1006/jmbi.1999.3383

[12] Daly N.L., Love S., Alewood P.F., Craik D.J. Chemical synthesis and folding pathways of large cyclic polypeptides: studies of the cystine knot polypeptide kalata B1. Biochemistry. 1999; 38:10606–10614. 10441158 10.1021/bi990605b

[13] Przytycki J.H., Traczyk P. Invariants of links of Conway type. 2016; arXiv:1610.06679. Preprint: not peer reviewed.

[14] Millett K.C., Rawdon E.J., Stasiak A., Sulkowska J.I. Identifying knots in proteins. Biochem. Soc. Trans. 2013; 41:533–537. 23514149 10.1042/BST20120339

[15] Rawdon E., Millett K., Sulkowska J., Stasiak A. Knot localization in proteins. Biochem. Soc. Trans. 2013; 41:538–541. 23514150 10.1042/BST20120329

[16] Dabrowski-Tumanski P., Stasiak A., Sulkowska J.I. In search of functional advantages of knots in proteins. PLoS One. 2016; 11:e0165986. 27806097 10.1371/journal.pone.0165986 PMC5091781

[17] Christian T., Sakaguchi R., Perlinska A.P., Lahoud G., Ito T., Taylor E.A., Yokoyama S., Sulkowska J.I., Hou Y.-M. Methyl transfer by substrate signaling from a knotted protein fold. Nat. Struct. Mol. Biol. 2016; 23:941. 27571175 10.1038/nsmb.3282 PMC5429141

[18] Craik D.J., Daly N.L., Waine C. The cystine knot motif in toxins and implications for drug design. Toxicon. 2001; 39:43–60. 10936622 10.1016/s0041-0101(00)00160-4

[19] Craik D.J., Simonsen S. L. D.N. The cyclotides: novel macrocyclic peptides as scaffolds in drug design. Curr. Opin. Drug Discov. Dev. 2002; 5:251–260. 11926131

[20] Jennings C., West J., Waine C., Craik D., Anderson M. Biosynthesis and insecticidal properties of plant cyclotides: the cyclic knotted proteins from Oldenlandia affinis. Proc. Natl. Acad. Sci. U.S.A. 2001; 98:10614–10619. 11535828 10.1073/pnas.191366898 PMC58514

[21] Jamroz M., Niemyska W., Rawdon E.J., Stasiak A., Millett K.C., Sulkowski P., Sulkowska J.I. KnotProt: a database of proteins with knots and slipknots. Nucleic Acids Res. 2015; 43:D306–D314. 25361973 10.1093/nar/gku1059 PMC4383900

[22] Dorier J., Goundaroulis D., Benedetti F., Stasiak A. Knoto-ID: a tool to study the entanglement of open protein chains using the concept of knotoids. Bioinformatics. 2018; 34:3402–3404. 29722808 10.1093/bioinformatics/bty365

[23] Turaev V. Knotoids. Osaka J. Math. 2012; 49:195–223.

[24] Rubach P., Sobolewski M. Dynamic SLA negotiation in autonomic federated environments. OTM Confederated International Conferences “On the Move to Meaningful Internet Systems”. 2009; Springer 248–258.

[25] Abramowicz M., Kamieniecki K., Piechna A., Rubach P., Piechna J. Using ANSYS and SORCER modeling framework for the optimization of the design of a flapping wing bionic object. Mach. Dyn. Res. 2016; 39.

[26] Dabrowski-Tumanski P., Sulkowska J. To tie or not to tie? That is the question. Polymers. 2017; 9:454. 10.3390/polym9090454 PMC6418553 30965758

[27] Inglis A.S., Liu T.-Y. The stability of cysteine and cystine during acid hydrolysis of proteins and peptides. J. Biol. Chem. 1970; 245:112–116. 5460796

[28] Trivedi M.V., Laurence J.S., Siahaan T.J. The role of thiols and disulfides on protein stability. Curr. Protein Pept. Sci. 2009; 10:614–625. 19538140 10.2174/138920309789630534 PMC3319691

[29] Dabrowski-Tumanski P., Jarmolinska A.I., Niemyska W., Rawdon E.J., Millett K.C., Sułkowska J.I. LinkProt: a database collecting information about biological links. Nucleic Acids Res. 2017; 45:D243–D249. 27794552 10.1093/nar/gkw976 PMC5210653

[30] Dabrowski-Tumanski P., Sulkowska J.I. Topological knots and links in proteins. Proc. Natl. Acad. Sci. U.S.A. 2017; 114:3415–3420. 28280100 10.1073/pnas.1615862114 PMC5380043

[31] de Oliveira A.L., Gallo M., Pazzagli L., Benedetti C.E., Cappugi G., Scala A., Pantera B., Spisni A., Pertinhez T.A., Cicero D.O. The structure of the elicitor Cerato-platanin (CP), the first member of the CP fungal protein family, reveals a double ψβ-Barrel fold and carbohydrate binding. J. Biol. Chem. 2011; 286:17560–17568. 21454637 10.1074/jbc.M111.223644 PMC3093830

[32] Hayashida S., Ohta K., Mo K. Cellulases of Humicola insolens and Humicola grisea. Methods Enzymol. 1988; 160:323–332.

[33] Xu B., Hellman U., Ersson B., Janson J. Purification, characterization and amino-acid sequence analysis of a thermostable, low molecular mass endo-β-1,4 glucanase from blue mussel, Mytilus edulis. Eur. J. Biochem. 2000; 267:4970–4977. 10931178 10.1046/j.1432-1327.2000.01533.x

[34] Craik D.J., Daly N.L., Bond T., Waine C. Plant cyclotides: A unique family of cyclic and knotted proteins that defines the cyclic cystine knot structural motif. J. Mol. Biol. 1999; 294:1327–1336. 10600388 10.1006/jmbi.1999.3383

[35] Gran L. Isolation of oxytocic peptides from Oldenlandia affinis by solvent extraction of tetraphenylborate complexes and chromatography on sephadex LH-20. Lloydia. 1973; 36:207–208. 4744557

